# Racial/Ethnic Differences in Perceived Access, Environmental Barriers to Use, and Use of Community Parks

**Published:** 2010-04-15

**Authors:** Susan A. Carlson, Joseph D. Brooks, David R. Brown, David M. Buchner

**Affiliations:** Centers for Disease Control and Prevention; Centers for Disease Control and Prevention, Atlanta, Georgia; Centers for Disease Control and Prevention, Atlanta, Georgia; Centers for Disease Control and Prevention, Atlanta, Georgia

## Abstract

**Introduction:**

Community parks provide places for people to be physically active. Our objective was to determine how access to, barriers to use of, and use of community parks differ by race/ethnicity.

**Methods:**

Analyses are based on a cross-sectional national sample of adults (N = 5,157) participating in the 2006 HealthStyles mail survey. Community parks were defined as outdoor public areas within 10 miles or a 20-minute drive from where a person lives that include walking/bike paths, nature preserves, playgrounds, beaches, lakes, rivers, or similar places.

**Results:**

Overall, 12% of respondents reported not having a community park. Among those with a community park, 14% reported personal safety concerns and 14% reported inadequate or poorly maintained facilities as barriers to park use. Race/ethnicity was not associated with park access; however, Hispanics and non-Hispanic blacks were more likely than non-Hispanic whites to report barriers. Among those with access to a community park, 83% reported any park use in the previous year and, of these, 67% reported an active visit. Odds of any park use did not differ significantly by race/ethnicity. Odds of an active visit were significantly lower in non-Hispanic blacks than whites (odds ratio, 0.67) but did not significantly differ between Hispanics and non-Hispanic whites.

**Conclusions:**

Parks are valuable community resources to all racial/ethnic groups. To promote and increase community park use, it is important to be aware that parks are used differently by different racial/ethnic groups and that barriers may differentially influence park use.

## Introduction

Despite the well-documented benefits of regular physical activity (1), most American adults fail to meet national health objectives for physical activity (2). The percentage of adults who fail to meet those objectives is higher in certain minority racial/ethnic groups than in whites (2). The *Guide to Community Preventive Services* has identified 8 strategies to increase physical activity, including "creation of or enhanced access to places for physical activity combined with informational outreach activities" (3). Community parks offer access to physical activity; recently, efforts have been made to bring the fields of public health and parks and recreation together to promote physical activity (4,5). The success of these efforts will depend in part on an understanding of how park access, environmental barriers to park use, and use of community parks differ by demographic characteristics, especially those characteristics in which disparities in physical activity exist, such as belonging to a minority racial/ethnic group.

Previous studies of racial/ethnic variations in access to places for physical activity have produced mixed results. Some studies have shown that people in minority-dominated communities have less access to recreational facilities than people in other neighborhoods and communities (6-9). Other studies have shown that minority communities have equal or more access to certain types of physical activity resources, including greenways and parks (9-11). Studies have also examined the racial/ethnic variation in frequency of park use (12-14) and active park use (for example, walking, playing sports, biking in a park) (13-17). Some studies have shown that minority groups are more likely to be active in parks (15,16), while other studies have found that minority groups are more sedentary park users (14,17). Park use may also be influenced by perceptions about environmental barriers, such as safety concerns and inadequate or poorly maintained facilities (18), and the influence of the barriers on park use may differ by race/ethnicity (19).

We focused on community parks, which were broadly defined to include all outdoor areas that respondents identified as a community park and that respondents perceived to be near where they live. The first purpose of this study was to determine how perceptions of community parks differed by race/ethnicity, including perceptions about community park access and environmental barriers to use. The second purpose was to determine how community park use in the past year (including frequency of use and types of activities engaged in) differed by race/ethnicity. The final purpose was to examine the association between perceived environmental barriers and park use (including any park use in the past year and active park use) and assess if these associations were consistent across racial/ethnic groups.

## Methods

### Survey and analytical sample

The survey data used in this study were obtained from the ConsumerStyles and HealthStyles databases managed by Porter Novelli, a public relations firm. The surveys are conducted annually in English and are designed to assess people's health-related attitudes, health behavior, consumer behavior, and media habits (20).

The sampling and data collection were conducted by Synovate, Inc (Chicago, Illinois). Synovate annually recruits approximately 450,000 households in the United States to be part of the Synovate mail panel survey, and participants agree to participate in periodic mail surveys in exchange for gifts, such as 30-minute telephone calling cards and a lottery chance to win $50 to $1,000 per completed survey.

From May through June 2006, the ConsumerStyles survey was mailed to a sample of 20,000 potential adult respondents who were selected through a stratified random sampling of the Synovate mail panel. The initial sample (N = 11,000) was stratified by region, household income, population density, age, and household size to create a nationally representative sample. A low-income/minority supplementary sample (N = 3,000) ensured adequate representation of these groups, and a households-with-children supplementary sample (N = 6,000) ensured adequate numbers of respondents for a follow-up survey focusing on children. Of the 20,000 households that were sent ConsumerStyles surveys, 13,260 (66%) returned the survey. The ConsumerStyles survey collected the demographic data used in the analysis.

From late June through early August 2006, the HealthStyles survey was sent to 6,600 randomly selected ConsumerStyles respondents. Of the 6,600 households that were sent the HealthStyles survey, 5,251 (80%) responded. Respondents whose questionnaires were missing data on frequency of park visits were excluded (n = 29). The final analytic sample size was 5,222 respondents. Questions about community parks were drafted by the Centers for Disease Control and Prevention and were included as part of the HealthStyles survey.

### Measures

Before being asked the survey questions related to community parks, all respondents were provided with the following definition: "A community park is an outdoor public area that is near to where you live and includes walking or bike paths, nature preserves, playgrounds, beaches, lakes, rivers, and similar places. The park should be within 10 miles or a 20-minute drive from where you live. Consider all parks whether they are city, state, or national parks."

To measure frequency of park use, respondents were asked how frequently during the previous year they had visited a community park: 4 to 7 days per week, 1 to 3 days per week, a few times per month, a few times per year, or never, and for this analysis responses were collapsed into 4 categories: weekly, monthly, yearly, and never. Respondents were classified as reporting any park use in the previous 12 months if they reported weekly, monthly, or yearly use.

To measure barriers to park use, respondents were asked to indicate which of the following things prevented them from using a community park: "my community does not have a park," "not enough time," "not enough money," "personal safety concern," "personal health problem," and "inadequate or poorly maintained facilities." Respondents were instructed to check all that apply. Because our study focus was on disparities in perceived accessibility and how accessibility relates to park use, we analyzed the 3 environmental barriers to use (no park in the community, personal safety concern, and inadequate or poorly maintained facilities).

To measure how active park users were, respondents were asked which of the following activities they participated in: walking/hiking, picnicking, relaxing, swimming, biking, running/jogging, attending an outdoor event, playing sports, and attending a gathering of family or friends. Respondents could identify multiple activities. An active visit was defined as reporting participation in any of the following activities: walking/hiking, swimming, biking, running/jogging, or playing sports.

### Statistical analysis

We examined prevalence of perceived barriers to park use, frequency of park use, and participation in certain activities in a park by race/ethnicity. Data were weighted to US census population projections for 2006 by sex, age, income, race/ethnicity, and household size. Significant differences in prevalence estimates by race/ethnicity were assessed by using pairwise *t* tests, and differences were considered significant at *P* < .05.

We conducted multivariate logistic regression analyses to examine the odds ratios by race/ethnicity (adjusted for sex, age, and household income) of reporting each environmental barrier, any park use, and any active park use (including additional adjustment for frequency of park use). Separate multivariate logistic regression models (adjusted for sex, age, and household income) were constructed to examine the association between each perceived barrier and any park use and active use. These models were stratified by race/ethnicity to examine if the influence of barriers on any park use and active use differed by race/ethnicity. To test for effect modification, the cross-product of each barrier and race/ethnicity was included in the regression model and was tested for significance by using an adjusted Wald *F* statistic. Analyses were conducted by using SUDAAN version 9.0 (RTI International, Research Triangle Park, North Carolina).

## Results

The demographic distribution of the unweighted sample differed slightly from that of the sample weighted to the US adult population (Table 1). The unweighted sample had a lower percentage of men and adults younger than 35 years than the weighted sample.

Only 12% of all respondents reported not having a community park as a barrier to use. This barrier did not differ significantly by race/ethnicity (Table 2). Among respondents with access to a community park, 14% reported personal safety concerns, 14% reported poorly maintained or inadequate facilities, and 6% reported both factors as barriers to park use. Adjusted odds of reporting either personal safety concerns or inadequate or poorly maintained facilities as being barriers to park use were higher among non-Hispanic blacks and Hispanics than among non-Hispanic whites (Table 2).

Among respondents with access to a community park, 43% reported yearly use, 25% monthly use, 15% weekly use, and 17% no use. The percentage who reported weekly park use was higher among Hispanics than non-Hispanic whites and was higher among respondents of "other" race/ethnicity than among non-Hispanic whites and non-Hispanic blacks (Table 3). Yearly park use was lower among those in the "other" race category than among non-Hispanic whites and blacks. When examining the odds ratios of any park use by race/ethnicity, no significant differences were observed (Table 3).

The association between reporting a personal safety concern as a barrier to park use and any park use was modified by race/ethnicity (Table 4). Non-Hispanic whites and blacks who reported a personal safety concern were significantly less likely to use a park than those who had not reported a personal safety concern; however, among Hispanics and those in the "other" race/ethnicity category, reporting a personal safety concern was not associated with park use. Respondents' perception that park facilities were inadequate or poorly maintained was not significantly associated with any park use in any racial/ethnic group.

We found racial/ethnic differences in participation in specific park activities (Figure). Non-Hispanic blacks were significantly less likely to report walking or hiking than non-Hispanic whites. Hispanics and respondents of "other" race/ethnicity were more likely to report running/jogging and playing sports than non-Hispanic whites. Non-Hispanic whites were less likely to attend a gathering of family or friends than non-Hispanic blacks.

**Figure.**
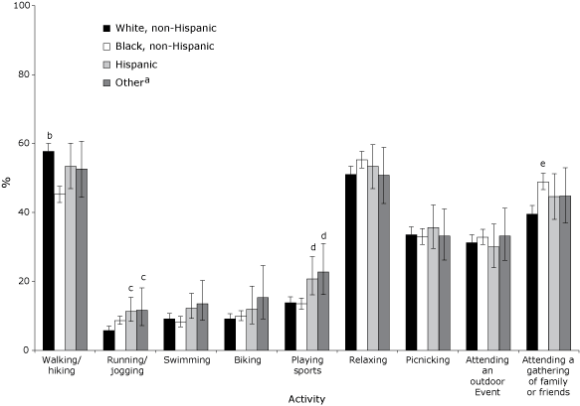
Participation in various activities during the previous 12 months among adult park visitors by race/ethnicity, HealthStyles 2006 (n = 3,763). Respondents were asked to indicate which of the following 9 activities that they had participated in: walking/hiking, picnicking, relaxing, swimming, biking, running/jogging, attending an outdoor event, playing sports, and attending a gathering of family or friends. Respondents could select multiple activities; 175 respondents did not select any. Error bars indicate 95% confidence intervals.

**Table:** 

**Activity**	**Frequency of participation, % (95% Confidence Interval)**			
	**White, non-Hispanic**	**Black, non-Hispanic**	**Hispanic**	**Other^a^**
Walking/hiking	58 (55-60)^b^	45 (39-52)	53 (48-59)	53 (44-61)
Running/jogging	6 (5-7)	9 (6-13)	11 (8-15)^c^	12 (7-18)^c^
Swimming	9 (8-11)	8 (5-12)	12 (9-17)^d^	13 (9-20)^d^
Biking	9 (8-11)	10 (6-17)	12 (8-17)	15 (9-25)
Playing sports	14 (12-15)	14 (9-20)	21 (16-26)	23 (16-31)
Relaxing	51 (49-53)	55 (49-62)	53 (47-59)	51 (43-59)
Picnicking	33 (31-36)	33 (27-40)	36 (30-41)	33 (26-41)
Attending an outdoor event	31 (29-34)	33 (27-39)	30 (25-36)	33 (26-41)
Attending a gathering of family or friends	40 (37-42)	49 (42-56)^e^	45 (39-51)	45 (37-53)

a “Other” race/ethnicity includes American Indian, Alaska Native, Asian, Native Hawaiian, and other Pacific Islander.

b In a pairwise comparison (t test), prevalence was significantly higher than in non-Hispanic blacks (non-Hispanic whites, P = .001, df = 2,979).

c In a pairwise comparison (t test), prevalence was significantly higher than in non-Hispanic whites (Hispanics, P = .003, df = 3,069; “other” race/ethnicity, P = .04, df = 2,804).

d In a pairwise comparison (t test), prevalence was significantly higher than in non-Hispanic whites (Hispanics, P = .01, df = 3,069; “other” race/ethnicity, P = .02, df = 2,804).

e In a pairwise comparison (t test), prevalence was significantly higher than in non-Hispanic whites (non-Hispanic blacks, P = .01, df = 2,979).

Overall 67% of park users reported at least 1 physically active park visit. Odds of an active visit were lower among non-Hispanic blacks than among non-Hispanic whites, but the odds did not significantly differ between Hispanics and non-Hispanic whites (Table 5). Compared with yearly park users, more frequent park users were more likely to report active park use in the previous year (weekly, adjusted odds ratio [AOR], 4.10 [95% confidence interval (CI), 3.04-5.51]; monthly, AOR, 2.95 [95% CI, 2.38-3.65]).

Reporting a barrier, either personal safety or inadequate/poorly maintained facilities, was not significantly associated with odds of an active visit among any racial/ethnic group (data not shown).

## Discussion

Most of the survey population perceived that they had access to a community park, and this percentage did not differ by race/ethnicity. Most people with access to a community park reported using it in the past year, and use did not differ by race/ethnicity, even though social (ie, safety concerns) and physical (ie, poor quality of facilities) environmental barriers to park use were more likely to have been reported by minority racial/ethnic groups. In addition, Hispanics were more likely than whites to be active park users. These results suggest that racial/ethnic disparities in physical activity cannot all be explained by disparities in access to community parks and active park use (2).

Prevalence of any park use did not vary significantly by race/ethnicity. In a sample limited to respondents from 2 metropolitan areas in the eastern United States (Atlanta, Georgia, and Philadelphia, Pennsylvania), Koreans, Hispanics, and whites were most likely and African Americans were least likely to visit a park in the previous year. In a nationwide sample, however, no significant association was found between race/ethnicity and park use (21).

We observed difference in the likelihood of an active park visit by race/ethnicity. The likelihood of an active visit was lower among non-Hispanic blacks than non-Hispanic whites, while non-Hispanic whites and Hispanics were as likely to have an active visit. The opposite effect was observed in Chicago park users: park users in African American neighborhoods were more likely than whites to be observed in physical activity (16). In another Illinois study, however, researchers found similar results to ours: non-Hispanic blacks were less likely to participate in active park activities, especially walking (14). Results among Hispanics were similar to other US studies that have found Hispanics to be more likely than non-Hispanic whites to participate in group sports (15,17). Other research has found that Hispanics were more likely to participate in sedentary activities (ie, picnicking, lounging in the grass, sitting on park benches) (17), but we observed no differences for Hispanics in the participation in more sedentary activities.

The association between personal safety concerns and any park use differed by race/ethnicity; non-Hispanic whites and blacks were less likely to use a park if they reported a safety concern. However, Hispanics were not less likely to use a park if they reported a safety concern. In an analysis restricted to 8 parks in minority communities, concerns about park safety did not predict park use (22). Other researchers have hypothesized that different racial groups may have different methods for coping with concerns, so barriers may have less of an influence on behavior (19). For example, parenting strategies implemented by African Americans, such as the use of kinship networks and neighborhood organizations, allowed some children in urban neighborhoods to participate in leisure activities available in their neighborhood, despite risks (crime, violence, limited resources) (23). Future research should examine how barriers may differentially affect park use among different racial/ethnic groups.

The percentage of respondents who reported that inadequate or poorly maintained facilities were a barrier to any park use differed by race/ethnicity. At least 2 potential reasons explain these differences: lack of resources or funding for parks in minority communities and differences in how different racial/ethnic groups use parks. An example of the latter is that Hispanics were more likely than non-Hispanic whites to report the status of park facilities as being a barrier to park use, perhaps because they are more likely than non-Hispanic whites to participate in group or individual sports that require specific facilities. This may also be in part why no association was observed between reporting the status of park facilities as being a barrier and park use. That is, park users may be more aware than nonusers of the status of the facilities because they use the park. The extent to which the presence and quality of specific facilities predict park use among various racial/ethnic groups should be addressed in future studies.

Study limitations included the sample selection bias associated with the use of data from a mail panel survey of volunteers. The sample was, however, drawn from a large, broad, community-dwelling population, and the demographic distribution of sample members was similar to US census population projections, except for differences caused by oversampling of low-income households and households with children.

Another limitation was lack of data on exact distance that respondents lived from a community park; such data may have helped us more precisely describe Americans' use of community parks and would have allowed us to examine the influence of proximity of the community park on our outcomes of interest. Previous studies of the association between environmental features and physical activity have used a variety of spatial referents (24), and our definition of a community as within 10 miles or a 20-minute drive has been used in studies that have found a correlation between participation in physical activity and access to community facilities (25,26).

Information on frequency and time spent doing each activity at a park was lacking, and respondents could select activities from a list of 9. We used a broad definition of an active park visit. Future studies would benefit from obtaining more information about specific activities park users participate in, as well as the intensity, frequency, and duration of their participation, to allow for a more precise definition of active community park use.

Finally, this study relied on survey respondents' reported perception of their access to community parks and barriers to park use. We had no information on the reliability or validity of our self-report measures. At least 1 study has shown that reported access may not reflect more objectively measured access (25). However, people's perceived access to parks or other recreational facilities at the very least may include their awareness of a park in a certain area and the ease of accessibility. The *Guide to Community Preventive Services* (3) recommends not only increased access to facilities but increased access together with informational outreach, thereby highlighting a person's awareness of and perceptions about a location, which a measure of perceived access may capture.

Our findings suggest that parks are valuable community resources across all segments of the population. Parks provide opportunities for physical activity and enhance the social fabric of the community through gatherings and picnics. As the fields of parks and recreation and public health continue to collaborate to promote physical activity in parks, personnel in both fields should be mindful of how parks are used by different racial/ethnic groups, and how potential barriers differentially influence park use, to appropriately customize parks and park programs for the target communities.

## Figures and Tables

**Table 1:** Characteristics of Participants in HealthStyles Survey, 2006 (N = 5,222)^a^

**Characteristic**	n	Unweighted %^b^	Weighted % (95% Confidence Interval)^b^
**Sex**			
Men	2,346	45	48 (47-50)
Women	2,876	55	52 (50-53)
**Age, y**			
18-24	161	3	13 (11-15)
25-34	667	13	18 (17-20)
35-44	1,317	25	20 (19-21)
45-54	1,275	24	19 (18-20)
55-64	828	16	14 (13-15)
≥65	974	19	16 (15-17)
**Race/ethnicity^c^**			
White, non-Hispanic	3,542	68	69 (68-71)
Black, non-Hispanic	622	12	12 (10-13)
Hispanic	711	14	13 (12-14)
Other	347	7	6 (6-8)
**Education level^d^**			
Less than high school graduate	351	7	6 (5-7)
High school graduate	1,387	27	26 (25-28)
Some college	1,926	37	38 (36-39)
College graduate	1,493	29	30 (29-32)
**Household income, $1,000**			
≤14.9	896	17	13 (12-14)
15.0-24.9	570	11	13 (12-15)
25.0-39.9	776	15	18 (16-19)
40.0-59.9	873	17	18 (17-19)
≥60.0	2,107	40	38 (36-40)

a 29 respondents who did not indicate how often they visited a park were excluded.

b Percentages may not total 100 because of rounding.

c "Other" race includes American Indian, Alaska Native, Asian, Native Hawaiian, and other Pacific Islander.

d 65 respondents were missing information on education level.

**Table 2:** Environmental Barriers to Community Park Use by Race/Ethnicity, HealthStyles Survey, 2006

Race/Ethnicity	Barrier					

	No Park in Community (n = 643)		Personal Safety Concern (n = 653)		Inadequate or Poorly Maintained Facilities (n = 647)	

	% (95% CI)	OR (95% CI)^a^	% (95% CI)	OR (95% CI)^a^	% (95% CI)	OR (95% CI)^a^
White, non-Hispanic	13 (11-14)	1.00 [Reference]	11 (10-12)	1.00 [Reference]	11 (10-13)	1.00 [Reference]
Black, non-Hispanic	11 (9-14)	0.83 (0.61-1.12)	26 (21-31)^b^,^c^	2.51 (1.85-3.39)	20 (16-25)^d^	1.66 (1.19-2.32)
Hispanic	12 (9-16)	0.91 (0.64-1.28)	21 (17-26)^b^,^c^	2.15 (1.56-2.95)	22 (18-28)^d^	1.88 (1.37-2.59)
Other^e^	9 (6-13)	0.68 (0.44-1.05)	15 (10-20)	1.46 (0.95-2.23)	22 (16-30)^d^	2.08 (1.37-3.15)

Abbreviations: CI, confidence interval; OR, odds ratio.

a Models adjust for sex, age, and household income level.

b In a pairwise comparison (*t* test), prevalence was significantly higher than in non-Hispanic whites (non-Hispanic blacks, *P* < .001, df = 3,635; Hispanics, *P* < .001, df = 3,733).

c In a pairwise comparison (*t* test), prevalence was significantly higher than in the "other" race/ethnicity category (non-Hispanic blacks, *P* = .002, df = 844; Hispanics, *P* = .0498, df = 942).

d In a pairwise comparison (*t* test), prevalence was significantly higher than in non-Hispanic whites (non-Hispanic blacks, *P* < .001, df = 3,635; Hispanics, *P* < .001, df = 3,733; "other" race/ethnicity, *P* = .002, df = 3,404).

e "Other" race/ethnicity includes American Indian, Alaska Native, Asian, Native Hawaiian, and other Pacific Islander.

**Table 3:** Frequency of Park Use Among Adults With Access to a Community Park by Race/Ethnicity, HealthStyles Survey, 2006 (n = 4,579)

Race/Ethnicity	Frequency of Park Use, % (95% CI)				Any Park Use, AOR (95% CI)^b^

	Weekly (n = 678)^a^	Monthly (n = 1,108)	Yearly (n = 1,977)	Never (n = 816)	
White, non-Hispanic	14 (12-15)	26 (24-28)	44 (42-46)^c^	17 (16-19)	1.00 [Reference]
Black, non-Hispanic	15 (12-20)	22 (17-27)	45 (39-50)^c^	18 (15-23)	0.91 (0.68-1.22)
Hispanic	21 (17-25)^d^	23 (19-27)	40 (35-46)	16 (13-20)	0.87 (0.66-1.16)
Other^e^	24 (17-32)^d^,^f^	26 (20-32)	34 (28-41)	16 (11-24)	0.88 (0.53-1.46)

Abbreviations: CI, confidence interval; AOR, adjusted odds ratio.

a Includes participants who responded 4-7 days/week or 1-3 days/week.

b Models adjust for sex, age, and household income level.

c In a pairwise comparison (*t* test), prevalence was significantly higher than in "other" race/ethnicity (non-Hispanic whites, *P* = .01, df = 3,404; non-Hispanic blacks, *P* = .02, df = 844).

d In a pairwise comparison (*t* test), prevalence was significantly higher than in non-Hispanic whites (Hispanics, *P* = .002, df = 3,733; "other" race/ethnicity, *P* = .009, df = 3,404).

e "Other" race/ethnicity includes American Indian, Alaska Native, Asian, Native Hawaiian, and other Pacific Islander.

f In a pairwise comparison (*t* test), prevalence was significantly higher than in non-Hispanic blacks ("other" race/ethnicity, *P* = .04, df = 1,173).

**Table 4:** Park Use in the Previous 12 Months and Barriers to Park Use by Race/Ethnicity, HealthStyles Survey, 2006 (n = 4,579)

Barrier	Park Use, AOR (95% CI)^a^				

	Overall	White, non-Hispanic	Black, non-Hispanic	Hispanic	"Other" race^b^
**Personal safety concern**					
No	1.00 [Reference]	1.00 [Reference]	1.00 [Reference]	1.00 [Reference]	1.00 [Reference]
Yes	0.69 (0.51-0.93)	0.60 (0.38-0.95)	0.41 (0.24-0.70)	1.24 (0.68-2.28)	2.28 (0.86-6.08)
**Inadequate or poorly maintained facilities**					
No	1.00 [Reference]	1.00 [Reference]	1.00 [Reference]	1.00 [Reference]	1.00 [Reference]
Yes	0.99 (0.72-1.34)	1.07 (0.76-1.52)	0.83 (0.44-1.58)	1.76 (0.83-3.72)	0.50 (0.19-1.31)

Abbreviations: AOR, adjusted odds ratio; CI, confidence interval.

a Adjusted Wald *P* value for interaction of race and personal safety concerns = .003; *P* value for interaction of race and inadequate or poorly maintained facilities = .19. Models adjust for sex, age group, and household income level.

b "Other" race/ethnicity includes American Indian, Alaska Native, Asian, Native Hawaiian, and other Pacific Islander.

**Table 5:** Likelihood of an Active Visit^a^ During the Previous 12 Months Among Adult Park Visitors by Race/Ethnicity, HealthStyles Survey, 2006 (n = 3,763)

**Race/Ethnicity**	≥1 Physically Active Park Visit (n = 2,477), % (95% CI)	AOR of Active Visit (95% CI)^b^
White, non-Hispanic	68 (66-70)^c^	1.00 [Reference]
Black, non-Hispanic	57 (50-64)	0.67 (0.49-0.91)
Hispanic	69 (63-74)^c^	1.00 (0.74-1.35)
Other^d^	72 (65-78)^c^	1.04 (0.72-1.50)

Abbreviations: CI, confidence interval; AOR, adjusted odds ratio.

a An active visit was defined as reported participation in walking/hiking, swimming, biking, running/jogging, or playing sports.

b Models adjust for sex, age, household income level, and frequency of park visits.

c In a pairwise comparison (*t* test), prevalence was significantly higher than in non-Hispanic blacks (non-Hispanic whites, *P* = .002, df = 2,979; Hispanics, *P* = .007, df = 957; "other" race/ethnicity, *P* = .002, df = 692).

d "Other" race/ethnicity includes American Indian, Alaska Native, Asian, Native Hawaiian, and other Pacific Islander.
